# Cerebrospinal fluid proteome alterations related to depressive symptoms in cognitive decline and Alzheimer's disease

**DOI:** 10.1002/alz.71054

**Published:** 2025-12-26

**Authors:** Miriam Rabl, Willem L. Hartog, Wiesje M. van der Flier, Yolande A. L. Pijnenburg, Charlotte E. Teunissen, Magda Tsolaki, Yvonne Freund‐Levi, Rik Vandenberghe, Lutz Froelich, Johannes Streffer, Simon Lovestone, Lars Bertram, Henrik Zetterberg, Johan Gobom, Stephanie J. B. Vos, Pieter Jelle Visser, Betty M. Tijms, Julius Popp

**Affiliations:** ^1^ Department of Psychiatry and Psychotherapy Psychiatric University Hospital Zurich University of Zurich Zurich Switzerland; ^2^ Alzheimer Center Amsterdam Department of Neurology Vrije Universiteit Amsterdam Amsterdam UMC Amsterdam the Netherlands; ^3^ Amsterdam Neuroscience Neurodegeneration Amsterdam UMC Amsterdam the Netherlands; ^4^ Epidemiology and Data Science Vrije Universiteit Amsterdam Amsterdam UMC location VUmc Amsterdam the Netherlands; ^5^ Alzheimer Nederland Amersfoort the Netherlands; ^6^ Amsterdam Public Health Amsterdam the Netherlands; ^7^ Neurochemistry Laboratory Department of Laboratory Medicine Amsterdam Neuroscience Vrije Universiteit Amsterdam Amsterdam UMC Amsterdam the Netherlands; ^8^ Department of Caregiver's Support Greek Association of Alzheimer's Disease and Related Disorders (GAADRD) Thessaloniki Greece; ^9^ Laboratory of Neurodegenerative Diseases Center for Interdisciplinary Research and Innovation (CIRI‐AUTH) Balkan Center Aristotle University of Thessaloniki Thessaloniki Greece; ^10^ Department of Clinical Science and Education Södersjukhuset Karolinska Institutet Stockholm Sweden; ^11^ School of Medical Sciences Öreebro University and Department of Psychiatry Örebro University Hospital Örebro Sweden; ^12^ Department of Neurology University Hospitals Leuven Leuven Belgium; ^13^ Laboratory for Cognitive Neurology KU Leuven Biomedical Sciences Group Leuven Belgium; ^14^ Department of Geriatric Psychiatry Central Institute of Mental Health Medical Faculty Mannheim University of Heidelberg Mannheim Germany; ^15^ H. Lundbeck A/S Valby Denmark; ^16^ Formerly Janssen R&D Beerse Belgium; ^17^ Department of Biomedical Sciences University of Antwerp Antwerp Belgium; ^18^ Department of Psychiatry University of Oxford Headington UK; ^19^ Johnson and Johnson Medical Ltd. Wokingham UK; ^20^ Lübeck Interdisciplinary Platform for Genome Analytics (LIGA) University of Lübeck Lübeck Germany; ^21^ Wisconsin Alzheimer's Disease Research Center School of Medicine and Public Health University of Wisconsin–Madison Madison Wisconsin USA; ^22^ Department of Psychiatry and Neurochemistry Institute of Neuroscience and Physiology the Sahlgrenska Academy at the University of Gothenburg Mölndal Sweden; ^23^ Clinical Neurochemistry Laboratory Hus V3 Sahlgrenska University Hospital Mölndal Sweden; ^24^ Department of Neurodegenerative Disease UCL Institute of Neurology London UK; ^25^ UK Dementia Research Institute at UCL London UK; ^26^ Hong Kong Center for Neurodegenerative Diseases Hong Kong China; ^27^ Department of Psychiatry and Neuropsychology Alzheimer Centrum Limburg School for Mental Health and Neuroscience Maastricht University Maastricht the Netherlands; ^28^ Department of Neurobiology Care Sciences and Society Division of Neurogeriatrics Karolinska Institutet Stockholm Sweden; ^29^ Old‐Age Psychiatry Service Department of Psychiatry University Hospital Lausanne Lausanne Switzerland

**Keywords:** Alzheimer's disease, amyloid pathology, cerebrospinal fluid, cholesterol, depression, neuropsychiatric symptoms, proteomics

## Abstract

**BACKGROUND:**

Depressive symptoms are common in cognitive decline and Alzheimer's disease (AD), but their underlying pathology remains poorly understood. We aimed to investigate the pathophysiology of depressive symptoms in the context of AD.

**METHODS:**

Individuals with normal cognition (NC), mild cognitive impairment (MCI), or mild AD dementia from two independent, large cohorts were included. Untargeted mass spectrometry–based cerebrospinal fluid (CSF) proteomics, regression analyses, and pathway‐enrichment analyses were applied.

**RESULTS:**

A total of 688 individuals (223 NC, 190 MCI, and 275 AD dementia) were included. The levels of 57 out of 946 robustly quantified CSF proteins were associated with depressive symptoms consistently across both cohorts. These proteins were enriched for cell adhesion/inflammation, synaptic signaling, and neurogenesis. In amyloid‐positive subjects, cholesterol metabolism and transport were additionally associated with depressive symptoms.

**CONCLUSION:**

The identified proteome alterations may reflect shared biological mechanisms involved in both AD and depression in older people.

**Highlights:**

- This is the first study to investigate cerebrospinal fluid proteome alterations associated with depressive symptoms in the context of cognitive decline and Alzheimer's disease (AD).
- Dysregulated proteins in patients with higher depression scores were related to pathways linked to cell adhesion/inflammation, synaptic signaling, and neurogenesis, as well as cholesterol metabolism in amyloid‐positive individuals.
- The identified proteome alterations may represent shared biological mechanisms involved in both AD and depression in older people.

## 1 BACKGROUND

Depressive symptoms are common in older people, and especially in individuals with cognitive decline and Alzheimer's disease (AD).^1^, ^2^, ^3^ Depressive symptoms are associated with lower quality of life and worse long‐term outcomes, such as earlier death and more rapid cognitive decline.^4^, ^5^ Nevertheless, little is known about the underlying pathological mechanisms involved.

Different pathophysiological alterations, including monoamine deficiency and proinflammatory processes, have been proposed to be involved in the pathogenesis of depression.^6^ Some studies have addressed the associations of depressive symptoms with AD pathology, particularly amyloid and tau pathology, providing inconsistent results.^7^, ^8^

Untargeted proteomics can be used to identify and quantify a large number of proteins in a single measurement, allowing for the molecular exploration of a wide spectrum of disease‐related changes without focusing a priori on specific molecular pathways or molecules.^9^, ^10^ While proteomics may represent an important approach to better understand the pathological mechanisms related to depressive symptoms, very few studies have investigated proteome changes associated with depressive symptoms. These studies performed proteomics in blood plasma, used small cohorts, or investigated major depressive disorder (MDD) in younger subjects.^11^, ^12^, ^13^, ^14^, ^15^ However, proteome alterations in blood plasma may substantially differ from those in cerebrospinal fluid (CSF),^16^ and alterations related to depression may differ in older people, particularly in the context of AD pathology. Only one study has investigated CSF proteomics using an untargeted mass spectrometry approach in relation to depression, conducted in a younger cohort of patients with MDD (*n* = 40 compared to *n* = 14 healthy controls; mean age of 48 years).^15^ Therefore, investigating CSF proteome changes in older individuals with and without cognitive impairment may provide more specific insights into the cerebral pathophysiological processes underlying depressive symptoms in aging and AD, while extending previous findings from younger participants with MDD.^17^

**FIGURE 1 alz71054-fig-0001:**
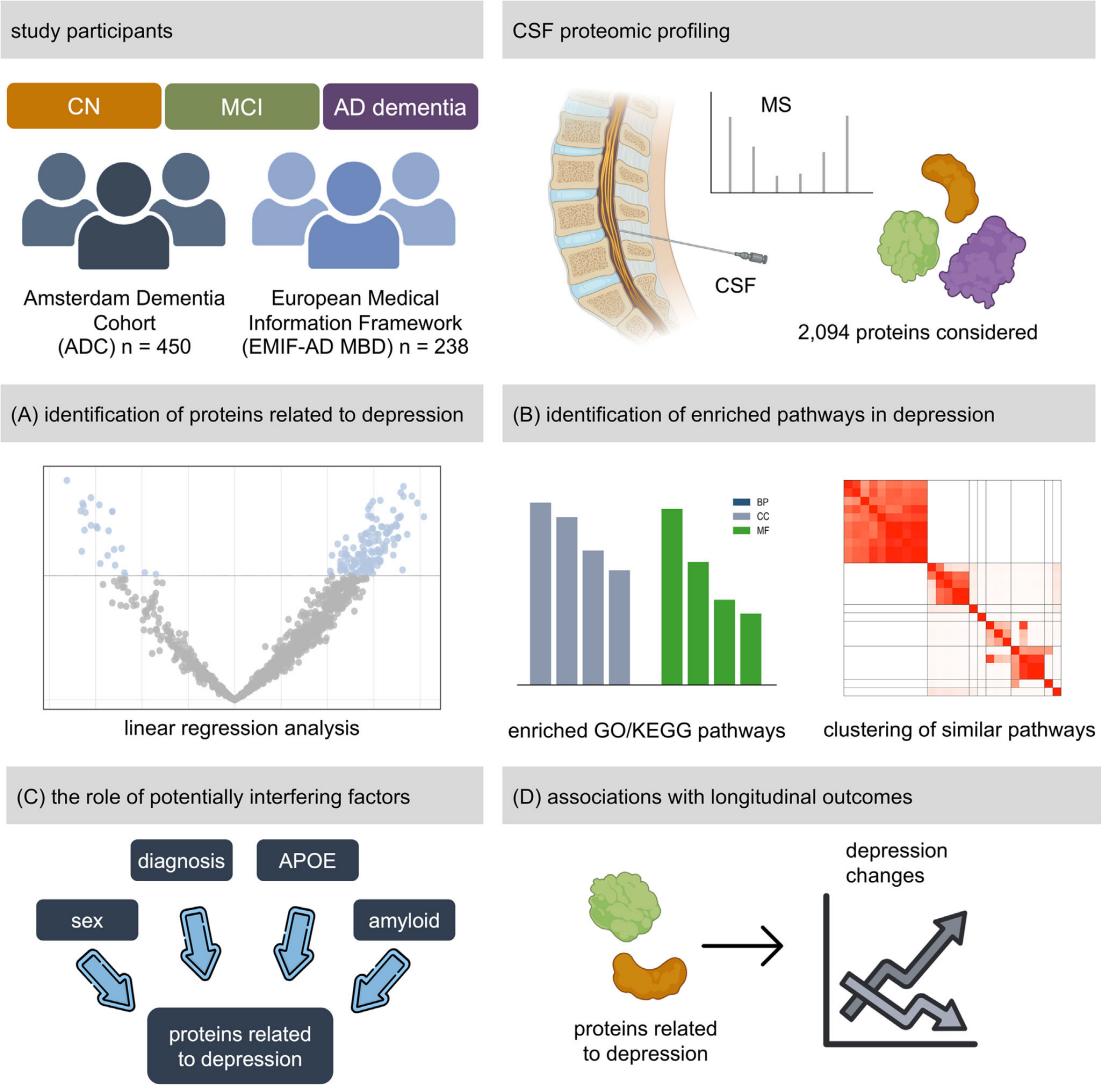
Study overview. We included 450 participants from the ADC and 238 from the EMIF‐AD MBD cohort. The subjects were diagnosed as CN, MCI, or mild AD dementia. Untargeted mass spectrometry–based proteomics in CSF was performed. A, We identified proteins associated with depressive symptoms via linear regression analysis with the GDS score as the dependent variable and all proteins as independent variables along with age and sex as covariates in both cohorts independently. B, Functional enrichment analysis was performed and enriched pathways were clustered for similarity. C, In addition, the influence of amyloid pathology, sex, and *APOE* ε4 status was investigated via stratified analysis. D, We also determined whether the selected proteins could predict longitudinal changes in depressive symptoms. AD, Alzheimer's disease; ADC, Amsterdam Dementia Cohort; *APOE*, apolipoprotein E; CN, cognitively normal; CSF, cerebrospinal fluid; EMIF‐AD MBD, European Medical Information Framework for Alzheimer's Disease Multimodal Biomarker Discovery study; GDS, Geriatric Depression Scale; GO, Gene Ontology; KEGG, Kyoto Encyclopedia of Genes and Genomes; MCI, mild cognitive impairment; MS, mass spectrometry.

A better understanding of the biological alterations underlying depressive symptoms, particularly in the presence of AD pathology, is essential for personalized diagnosis and monitoring and for developing new treatments for depression in the context of cognitive decline and AD. Here, we investigated the molecular and pathophysiological alterations related to depressive symptoms via an untargeted CSF proteomics approach in two large AD cohorts. Furthermore, we addressed the influence of amyloid pathology on proteome alterations and associations of proteins with changes in depressive symptoms over time (Figure 1).

## 2 METHODS

### 2.1 Study participants

Participants from two distinct cohorts, the single‐center Amsterdam Dementia Cohort (ADC) and the multi‐center European Medical Information Framework for Alzheimer's Disease Multimodal Biomarker Discovery study (EMIF‐AD MBD), were included. The ADC is a longitudinal, memory‐clinic cohort study aiming to identify new diagnostic and treatment strategies for dementia.^18^ The EMIF‐AD MBD is a consortium study that includes different European prospective AD cohorts aiming to discover novel biomarkers of AD and to unravel the pathophysiological mechanisms of AD.^19^ Participants from both cohorts were considered on the basis of the availability of CSF proteomics data and self‐rated depression scales performed within 3 months of CSF collection. Both cohorts included individuals at different clinical stages, such as normal cognition (NC), mild cognitive impairment (MCI), and mild AD dementia, and were recruited from university hospital memory clinics. Diagnostic criteria for MCI and AD dementia were used according to international consensus criteria.^18^, ^19^, ^20^ The exclusion criteria were any neurological disease other than suspected AD (i.e., Parkinson's disease, dementia with Lewy bodies, or vascular dementia) and/or any psychiatric or somatic disorders that could cause cognitive impairment.

**RESEARCH IN CONTEXT**

1. **Systematic review**: The authors reviewed the literature using traditional sources (e.g., PubMed) as well as meeting abstracts and presentations. Although the exact relationship between depression and Alzheimer's disease (AD) pathology remains unclear, emerging evidence suggests potential common pathophysiological mechanisms. However, no previous study has examined cerebrospinal fluid (CSF) proteome alterations related to depressive symptoms in cognitive decline and AD.
2. **Interpretation**: We identified 57 CSF proteins consistently associated with depressive symptoms in two independent cohorts. These proteins were enriched for cell adhesion/inflammation, synaptic signaling, and neurogenesis. Among amyloid‐positive subjects, additional enrichment in cholesterol metabolism and transport was observed. The identified proteome alterations may reflect both AD‐related and independent biological mechanisms underlying depressive symptoms in older people.
3. **Future directions**: Further research is needed to clarify the causes and consequences of these dysregulated processes associated with depressive symptoms, with particular attention to the contribution of AD pathology.

To assess AD pathology, amyloid beta (Aβ) pathology was defined based on the presence of an abnormal CSF Aβ marker. In the ADC, abnormal Aβ status was determined via a drift‐corrected cutoff of Aβ42 < 813 pg/mL.^21^ In EMIF‐AD MBD, abnormal Aβ status was defined by a centrally measured cutoff of CSF Aβ42/Aβ40 <  0.061. This cutoff was determined based on mixture model analyses comparing cognitively unimpaired and AD dementia groups in the total EMIF‐AD MBD dataset.^19^ CSF Aβ42 and Aβ40 levels were measured via an enzyme‐linked immunosorbent assay (ELISA) from Innotest (Fujirebio, formerly Innogenetics) in both cohorts.

### 2.2 Depressive symptoms

We used the 15‐item Geriatric Depression Scale (GDS), which was available for all included participants from the ADC and EMIF‐AD MBD. This questionnaire has been validated and widely used in memory clinics.^22^ The GDS consists of 15 items, each scored 1 point if related to depressive symptoms, whereas a total score > 4 indicates the presence of depression.^23^, ^24^ We used the linear GDS score to capture the severity of depressive symptoms. The raw values of the total GDS scores were normalized to approximate normality.[Fig alz71054-fig-0001]

### 2.3 CSF proteomics data

**FIGURE 2:**
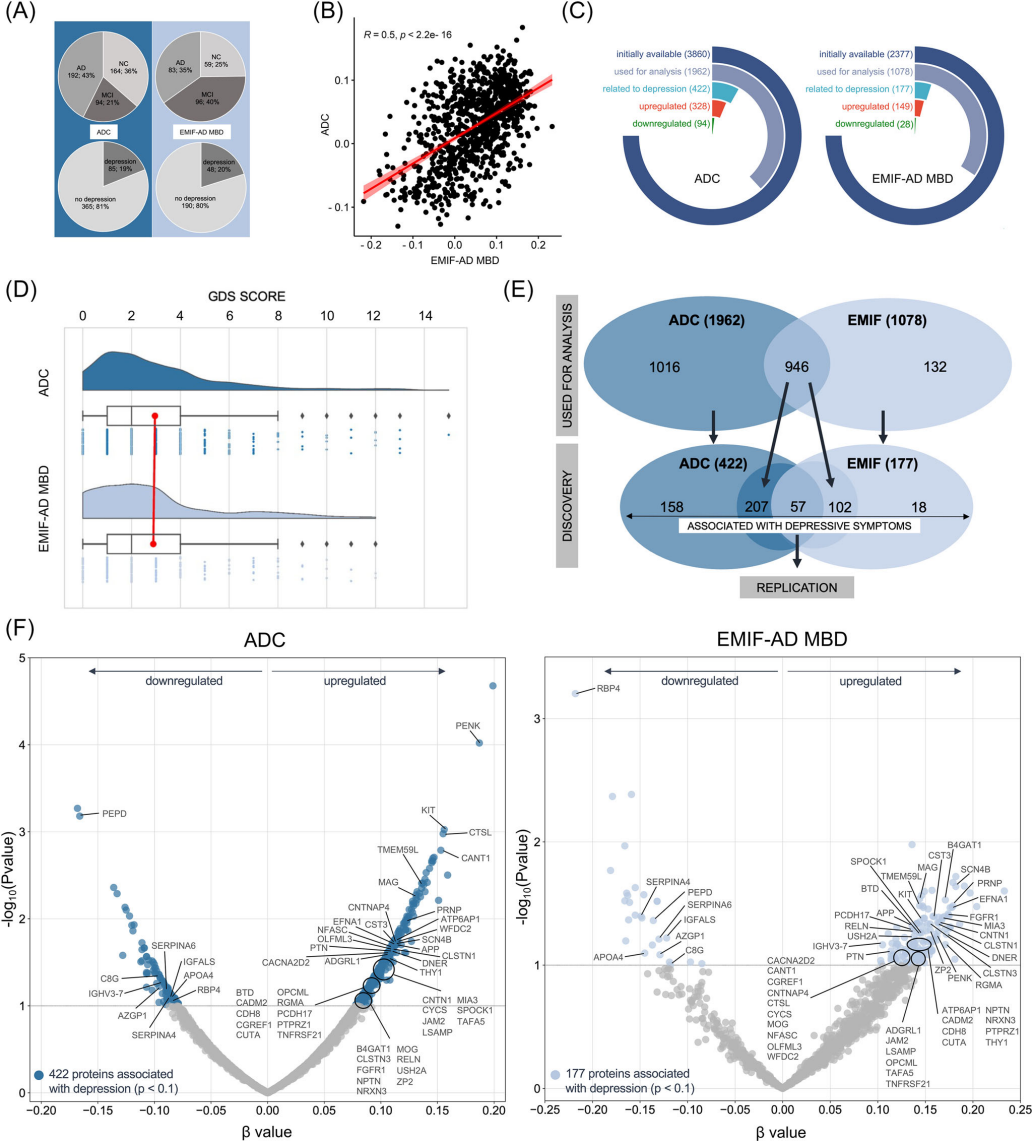
Cohort characteristics and selection of proteins associated with depressive symptoms in the ADC and EMIF‐AD MBD. A, Distribution of clinical diagnosis groups and frequency of depression (defined as GDS score > 4). B, Correlation plot showing the effect size (*x* axis) of 946 proteins in relation to the GDS score for the ADC and EMIF‐AD MBD. C, Number of proteins used for each step of selection. D, Raincloud plot showing the distribution of GDS scores. E, Selection process of proteins associated with depressive symptoms. F, Volcano plot showing the beta values (effect size) for each of the 946 proteins available in both cohorts (*x* axis). The *y* axis shows the *p* values for each protein, whereas a cutoff of *p* < 0.1 (not false discovery rate corrected) was used to select proteins associated with depressive symptoms in both cohorts. The selected proteins are colored, and the 57 replicated proteins are labeled with protein names. ADC, Amsterdam Dementia Cohort; EMIF‐AD MBD, European Medical Information Framework for Alzheimer's Disease Multimodal Biomarker Discovery study; GDS, Geriatric Depression Scale.

Tandem mass spectrometry–based measurement of proteomics data and preprocessing, including quality control and normalization of the CSF proteomic data for EMIF‐AD MBD and ADC, were performed as described in detail previously.^18^, ^25^, ^26^ In brief, protein abundances were corrected for technical variation between 16‐plex experiments using the internal reference scaling procedure or tandem mass tag (TMT) data, which uses a pool of CSF from all samples in two channels from each experiment.^27^ This two‐step normalization aligned total protein intensities across channels within each experiment and then applied correction factors based on pooled internal standards to harmonize values between TMT batches. Proteins lacking internal standards were excluded from further analyses. After batch correction, protein abundance values were log2‐transformed and harmonized between cohorts by scaling each protein relative to the mean and standard deviation levels of a control group so that positive and negative values were greater than or lower than the control group mean. The control group was defined as having no objective cognitive impairment, being amyloid negative, and having no depression (GDS score < 5). This resulted in 87 available controls for the ADC and 34 controls for the EMIF‐AD MBD. This approach should optimize comparability between the cohorts so that the protein averages for both cohorts are similar and comparable when performing regression analysis (Figure 2B). A total of 3860 proteins in the ADC and 2377 in the EMIF‐AD MBD, of which 2014 overlapped, were initially available. Only proteins observed in > 75% of the samples were considered for analysis (*n* = 1962 in ADC, *n* = 1078 in EMIF‐AD MBD, of which 946 overlapped and were considered “robustly quantified”, see also Figure 2C). For all proteins, we report the gene names and UniProt numbers.

### 2.4 Statistical analysis

For the descriptive statistics, SPSS (IBM, V.28.0) was used. Other analyses were performed via R version 4.4.1 including the “emmeans”^28^ and “simplifyEnrichment”^29^ packages. *T* tests and Mann–Whitney *U* tests were performed for continuous variables, and Pearson *χ* ^2^ test was used for categorical variables. The Shapiro–Wilk test was applied to assess the normality of all continuous variables. Based on the distribution, *t* tests were used for normally distributed variables, and Mann–Whitney *U* tests were applied for non‐normally distributed variables. Pearson χ^2^ test was used for categorical variables. To consider the false discovery rate (FDR), the Benjamini–Hochberg method was used to adjust the *p* values.^30^ For all the statistical tests, two‐tailed tests were used, and the alpha value was set at 0.05.

#### 2.4.1 Identification of proteins associated with depressive symptoms

To identify proteins associated with depressive symptoms, we applied linear regression analysis using the normalized GDS score (i.e., severity of depressive symptoms) as the dependent variable. Each protein was used in separate analyses as an independent variable along with age and sex as covariates. The identification of proteins associated with depressive symptoms was conducted separately in both cohorts (discovery), using a *p* value threshold of < 0.1 (not FDR corrected). Only proteins meeting these criteria in both cohorts (replication) were selected for further analysis. The selected proteins associated with depressive symptoms were then annotated to their corresponding biological processes according to the Panther database to provide functional insight into individual proteins associated with depressive symptoms. To investigate interactions between the proteins associated with depressive symptoms, protein–protein interaction (PPI) analysis was performed via the STRING database (https://string‐db.org/, accessed on November 23, 2024).^31^ The parameters included medium confidence scores (0.4) for interactions and five predicted functional partners on the basis of available evidence.

We additionally performed analyses using a categorical definition for the presence of depression (GDS > 4, dependent variable) by applying a binary logistic regression approach to identify proteins related to more severe GDS scores.^24^ Each protein was used in separate analyses as an independent variable along with age and sex as covariates.

#### 2.4.2 Identification of enriched pathways associated with depressive symptoms

To identify key pathways that may play an important role in depressive symptoms, we performed pathway enrichment analysis with the ShinyGO gene set enrichment tool V.082 of the South Dakota State University.^32^ We used the Gene Ontology (GO) and the Kyoto Encyclopedia of Genes and Genomes (KEGG) databases to investigate overrepresented pathways of the selected proteins. Because the GO enrichment analysis produces a long list of enriched pathways with highly redundant information, we applied the binary cut algorithm on semantic similarity scores^33^ to cluster functional terms, that is, gene products, into semantically similar groups via the simplifyGO function (simplifyEnrichment R package).^29^ We display all enriched pathways (FDR‐adjusted *p* < 0.05) in similarity heatmaps and report all identified pathways in detail in in the supporting information.

#### 2.4.3 Investigation of the influence of amyloid pathology, sex, clinical diagnosis, apolipoprotein E ε4 status, and age

Given that amyloid pathology may contribute to the manifestation of depressive symptoms^7^, ^8^, ^34^ we investigated the potential interaction effect of amyloid status on the relationship between individual proteins and depressive symptoms (GDS scores). Therefore, we included an additional interaction term with amyloid status and protein levels in our regression model and used marginal means (emmeans in R^28^) to estimate the effect of the protein levels on depressive symptoms in the presence/absence of amyloid pathology. Additionally, we repeated the analysis to detect proteins and enriched pathways stratified according to amyloid status, sex, clinical diagnosis, and apolipoprotein E (*APOE*) ε4 status. We also evaluated the association between proteins and age using Pearson correlation analysis.

#### 2.4.4 Investigation of longitudinal associations

We applied regression analysis to determine whether the selected proteins associated with depressive symptoms at baseline (independent variable) could predict a change in depressive symptoms or future depression (dependent variables). Changes in depressive symptoms were addressed via the GDS score change per year (the baseline GDS score was subtracted from the GDS score at follow‐up [FU] and divided by time to FU in years). A positive GDS change indicated a worsening of depressive symptoms. Future depression (binary definition) was defined as a GDS score > 4 at FU. Whenever multiple FU visits were available, we chose the visit closest to 1 year after the baseline visit for each subject.

## 3 RESULTS

### 3.1 Sample description

We included 450 individuals from the ADC (Table 1) and 238 from the EMIF‐AD MBD study (Table 2). The cohort characteristics according to the diagnostic group are shown in Table S1 in supporting information. While the distributions of the three diagnosis groups (cognitively unimpaired [NC], MCI, and mild AD dementia) were different, the number of depressed subjects (20% in the ADC group and 19% in the EMIF‐AD MBD group) and the mean total GDS score (3.0 in the ADC group and 2.9 in the EMIF‐AD MBD group) were similar in both cohorts (see Figure 2A, D). The ADC cohort consisted of younger participants and fewer females. The Shapiro–Wilk test indicated that only age was normally distributed, while the other continuous variables were non‐normally distributed.

**TABLE 1:** Characteristics of the ADC study participants with and without depression.

	Total *n* = 450	Abnormal GDS (GDS > 4) *n* = 85	Normal GDS (GDS < 5) *n* = 365	*p* value
**Demographic data**				
Female, *n* (%)	185 (41.1)	38 (44.7)	147 (40.3)	0.465^a^
Age, mean ± SD	63.6 ± 8.1	62.8 ± 8.0	63.8 ± 8.1	0.315^b^
Education years, mean ± SD	11.7 ± 3.0	11.1 ± 2.8	11.8 ± 3.0	**0.045** ^c^
**Diagnosis, *n* (%)**				
NC	164 (36.4)	33 (38.8)	131 (35.9)	0.619^a^
MCI	94 (20.9)	18 (21.2)	76 (20.8)	0.523^a^
AD dementia	192 (42.7)	34 (40.0)	158 (43.3)	0.627^a^
**Clinical data, mean ± SD**				
GDS	3.0 ± 2.6	7.3 ± 2.5	2.0 ± 1.3	**<0.001** ^c^
MMSE	24.9 ± 4.4	25.2 ± 4.2	24.9 ± 4.5	0.655^c^
time to FU	1.7 ± 1.7	2.1 ± 2.7	1.6 ± 1.4	0.256^c^
Yearly GDS change	−0.4 ± 2.3	−1.6 ± 2.8	−0.04 ± 2.1	**<0.001** ^c^
Yearly MMSE change	−0.4 ± 3.0	−0.5 ± 2.8	−0.4 ± 3.1	0.590^c^
**Biomarker, *n* (%)**				
Positive Amyloid status	339 (75.3)	61 (71.8)	278 (76.2)	0.404^a^
*APOE* ε4 carrier	250 (58.0)	45 (56.3)	205 (58.4)	0.802^a^

Abbreviations: ADC, Amsterdam Dementia Cohort; *APOE*, apolipoprotein E; FU, follow‐up; GDS, Geriatric Depression Scale; MCI, mild cognitive impairment; MMSE, Mini‐Mental State Examination; NC, normal cognition; SD, standard deviation.

^a^ Chi‐squared test.

^b^ *t* test.

^c^ Mann–Whitney *U* test.

**TABLE 2 alz71054-tbl-0002:** Characteristics of the EMIF‐AD MBD study participants with and without depression.

	Total *n* = 238	Abnormal GDS (GDS > 4) *n* = 48	Normal GDS (GDS < 5) *n* = 190	*p* value
**Demographic data**				
Female, *n* (%)	121 (50.8)	31 (64.6)	90 (47.4)	**0.037** ^a^
Age, mean ± SD	67.4 ± 7.6	66.4 ± 7.4	67.6 ± 7.7	0.331^b^
Education years, mean ± SD	10.7 ± 3.7	10.0 ± 3.6	10.8 ± 3.7	0.148^c^
**Diagnosis, *n* (%)**				
NC	59 (24.8)	7 (14.6)	52 (27.4)	0.091^a^
MCI	96 (40.3)	22 (45.8)	74 (38.9)	0.413^a^
AD dementia	83 (34.9)	19 (39.6)	64 (33.7)	0.499^a^
**Clinical data, mean ± SD**				
GDS	2.9 ± 2.7	7.4 ± 2.0	1.8 ± 1.3	**< 0.001** ^c^
MMSE	25.4 ± 3.8	24.9 ± 3.8	25.5 ± 3.8	0.198^c^
Time to FU	2.6 ± 1.4	2.8 ± 1.4	2.5 ± 1.3	0.292^c^
Yearly GDS change	−0.4 ± 1.9	−2.1 ± 2.7	0.5 ± 1.4	**0.017** ^c^
Yearly MMSE change	−0.8 ± 2.2	−0.7 ± 1.7	−0.9 ± 2.3	0.547^c^
**Biomarker, *n* (%)**				
Positive amyloid status	146 (61.3)	27 (56.3)	119 (62.6)	0.507^a^
*APOE* ε4 carrier	134 (56.3)	25 (52.1.0)	109 (57.4)	0.419^a^

Abbreviations: *APOE*, apolipoprotein E; EMIF‐AD MBD, European Medical Information Framework for Alzheimer's Disease Multimodal Biomarker Discovery study; FU, follow‐up; GDS, Geriatric Depression Scale; MCI, mild cognitive impairment; MMSE, Mini‐Mental State Examination; NC, normal cognition; SD, standard deviation.

^a^ Chi‐squared test.

^b^ *t* test.

^c^ Mann–Whitney *U* test.

### 3.2 Proteins and protein‐related pathways associated with depressive symptoms

Among the analyzed proteins (1962 in the ADC and 1078 in the EMIF‐AD MBD), 946 were identified in both cohorts. We observed a moderately strong correlation (*r* = 0.505, *p* < 0.001, 95% confidence interval [CI] 0.456, 0.551) of effect sizes for each protein and its relationship to depressive symptoms when the two cohorts were compared (Figure 2B). These findings suggest good consistency in the associations of proteins with depressive symptoms between the two cohorts.

We first identified 422/1962 proteins associated with depressive symptoms in the ADC (Table S2 in supporting information) and 177/1078 in the EMIF‐AD MBD (Table S3 in supporting information). We discovered 57 proteins (out of 946) associated with depressive symptoms that could be replicated in both cohorts (Figure 2E and Table S4 in supporting information). Among these proteins, 48 had higher levels with higher depression scores in both cohorts (upregulated), and 8 had lower CSF levels with higher depression scores in both cohorts (downregulated). One protein showed opposite regulatory patterns: being upregulated in EMIF‐AD MBD and downregulated in ADC (Figure 2F).

**FIGURE 3 alz71054-fig-0003:**
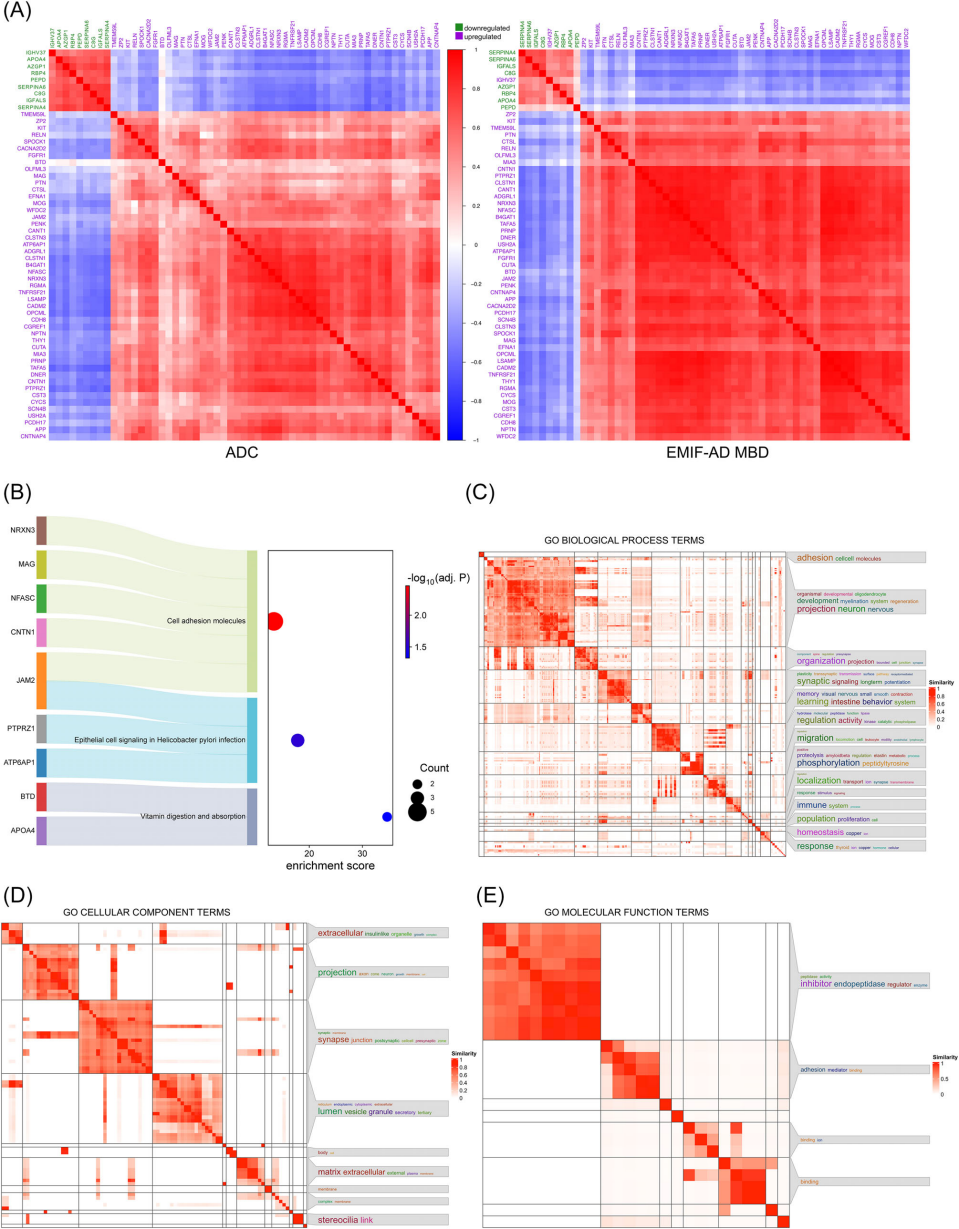
Characterization of 57 proteins associated with depressive symptoms. A, Heatmap showing the coexpression of the 57 proteins associated with depressive symptoms in both cohorts (ADC and EMIF‐AD MBD) via Pearson correlation analysis. Up‐ and downregulated proteins are labeled in violet and green, respectively. B, Enriched KEGG pathways associated with depressive symptoms. C‐E, Enriched GO pathways associated with depressive symptoms. Amsterdam Dementia Cohort; EMIF‐AD MBD, European Medical Information Framework for Alzheimer's Disease Multimodal Biomarker Discovery study; GO, Gene Ontology; KEGG, Kyoto Encyclopedia of Genes and Genomes.

Figure 3A shows the coexpression of the 57 proteins associated with depressive symptoms in both cohorts. The selected proteins included amyloid precursor protein (APP), cystatin C (CST3), peptidase D (PEPD), retinol binding protein 4 (RBP4), proenkephalin (PENK), and proto‐oncogene cKIT and were associated with cell adhesion, synaptic signaling, and neurogenesis (for the KEGG results, see Figure 3B and for the GO results, see Figure 3C‐E). The complete lists of the enriched KEGG and GO pathways are provided in Tables S5–S8 in supporting information. Additional information on all proteins found to be associated with depressive symptoms in either cohort converged on similar biological pathways (Figure S1A in supporting information). The network of protein–protein interactions of the 57 selected proteins associated with depressive symptoms is displayed in Figure S1B and revealed that especially APP shows high interconnectivity between the selected proteins.

#### 3.2.1 Proteins associated with depression (GDS > 4)

Additional analysis revealed that 1 of 57 proteins in EMIF‐AD MBD (RBP4 with odds ratio [OR] = 0.54 [0.37–0.77] and *p* < 0.001) and 5 of 57 proteins in ADC (CANT1 with OR 1.39 [1.07–1.81] and *p* = 0.014, PENK with OR = 1.35 [1.04–1.74] and *p* = 0.022, myelin‐associated glycoprotein [MAG] with 1.32 [1.04–1.69] and *p* = 0.025, olfactomedin‐like protein 3 [OLFML3] with OR = 1.30 [1.01–1.67] and *p* = 0.036, and sodium channel β‐subunit 4 [SCN4B] with OR 1.30 [1.01–1.67] and *p* = 0.038) were also associated with the presence of depression (defined as GDS > 4), but lost significance after FDR correction. Results on associations between depression (GDS > 4) and CSF proteins are shown in Tables S9–S10 in supporting information.

### 3.3 Influence of amyloid pathology, sex, clinical diagnosis, *APOE* ε4 status, and age

To further investigate the potential moderating effect of amyloid status on the relationship between individual proteins and depressive symptoms we performed interaction analyses including amyloid status. Among all 946 proteins tested, only the relationship between prodynorphin (PDYN) and depressive symptoms, was influenced by amyloid status across both cohorts (Tables S2–S3) and was not included in the 57 proteins associated with depressive symptoms across both cohorts. Lower PDYN levels in amyloid‐negative individuals and higher PDYN levels in amyloid‐positive individuals were associated with depressive symptoms.

**FIGURE 4 alz71054-fig-0004:**
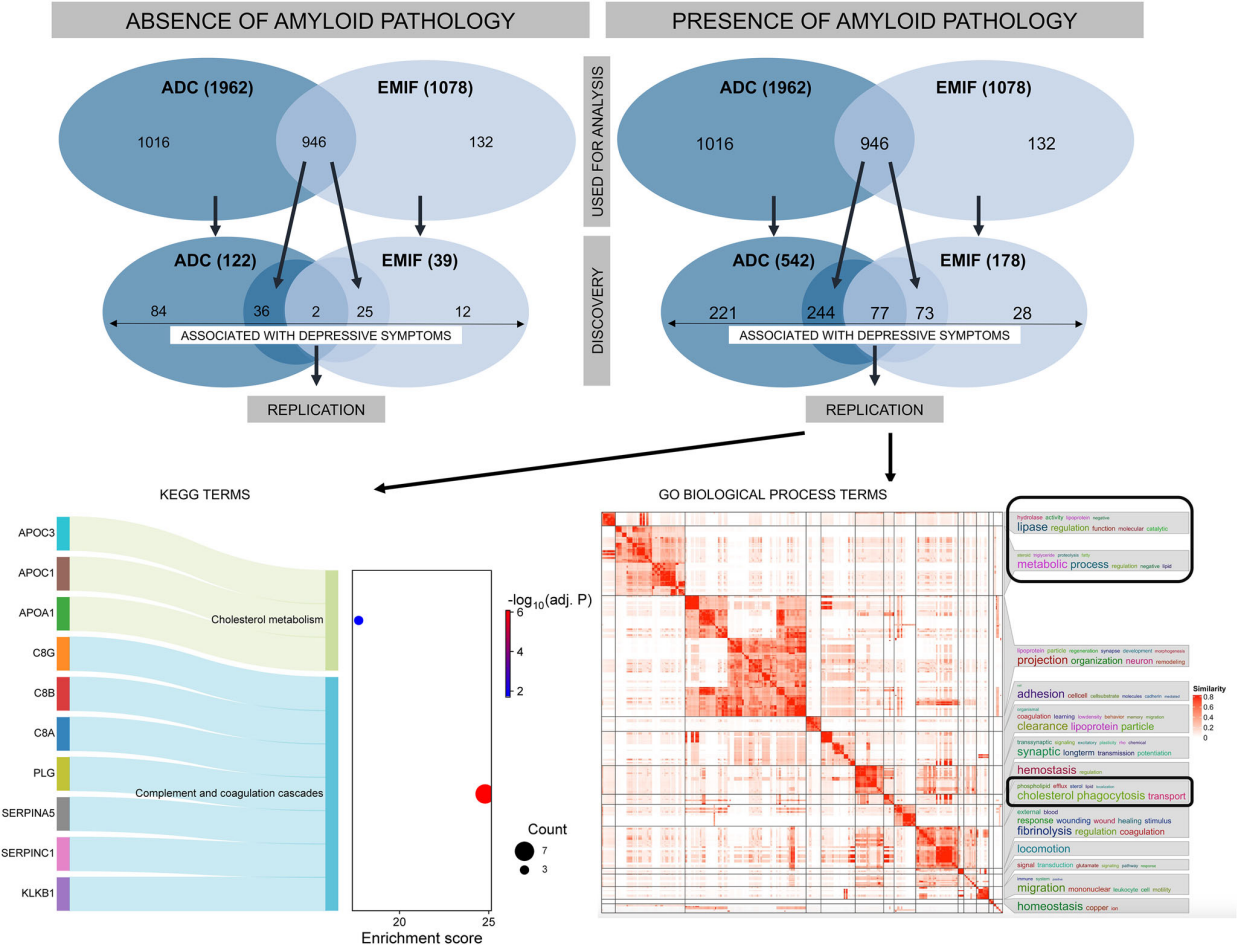
Proteins and pathways associated with depressive symptoms stratified according to amyloid status. Selection of proteins associated with depressive symptoms in the absence and presence of amyloid pathology. Enriched KEGG and GO pathways for amyloid‐positive individuals are shown. Pathways related to metabolic processes in amyloid‐positive individuals are highlighted in black on the right side. ADC, Amsterdam Dementia Cohort; EMIF‐AD MBD, European Medical Information Framework for Alzheimer's Disease Multimodal Biomarker Discovery study; GO, Gene Ontology; KEGG, Kyoto Encyclopedia of Genes and Genomes.

The results stratified according to amyloid status are shown in Figure 4 and Tables S2, S3, and S11 in supporting information. In amyloid‐positive individuals 77 proteins were associated with depressive symptoms, whereas in amyloid‐negative individuals, only two proteins were associated with depressive symptoms. Compared to the findings of the total cohort (*n* = 57 proteins associated with depressive symptoms) 23 proteins were also associated with depressive symptoms in amyloid‐positive individuals only. We performed pathway enrichment analysis and found similar enriched pathways in the total cohort and the amyloid‐positive group. In addition to neurogenesis, cell adhesion/inflammation, and synaptic signaling, cholesterol metabolism, and especially lipoproteins (APOA1, APOC1, APOC3, APOF), were associated with depressive symptoms when amyloid pathology was present. The complete lists of the enriched KEGG and GO pathways are provided in Tables S12–S15 in supporting information.

The results stratified by sex are shown in Figure S2 in supporting information and Tables S2, S3, and S11. In females, only three proteins were associated with depressive symptoms, whereas in males, we identified 30 proteins with several enriched pathways.[Fig alz71054-fig-0003], [Fig alz71054-fig-0004]

The results stratified for clinical diagnosis are shown in Tables S2, S3, and S11. In the NC group, 5 proteins; in the MCI group, 6 proteins; and in the clinical AD dementia group, 17 proteins were associated with depressive symptoms. The results stratified by *APOE* ε4 status are shown in Figure S3 in supporting information and Tables S2, S3, and S11. In *APOE* ε4 non‐carriers, only 3 proteins were associated with depressive symptoms, whereas in *APOE* ε4 carriers, we identified 14 proteins.

Of the 57 proteins associated with depressive symptoms, 3 were also associated with age in EMIF‐AD MBD and 4 in ADC after FDR correction. Only reelin (RELN) and corticosteroid‐binding globulin (SERPINA6) were associated with age in both cohorts. Full data on protein associations with age are shown in Tables S16–S17 in supporting information.

### 3.4 Associations with changes in depression scores over time

GDS at the FU was available for 236/450 (52.4%) subjects in the ADC and 42/238 (17.6%) in the EMIF‐AD MBD. Comparing subjects with depression (GDS > 4) to those without depression at baseline, subjects with depression presented a decrease of depression scores over time, whereas subjects without depression presented an increase in GDS scores in the EMIF‐AD MBD and remained stable in the ADC (Table 1 and Table 2). None of the 57 selected proteins associated with depressive symptoms at baseline were associated with the presence of depression or depression severity at FU (GDS > 4 or GDS score) or with changes in GDS scores over time, which was consistent across both cohorts (Tables S18–S19 in supporting information). Furthermore, none of the 77 proteins associated with depressive symptoms in amyloid‐positive subjects were associated with any of the mentioned longitudinal outcomes (data not shown).

## 4 DISCUSSION

Using a data‐driven CSF proteomics approach in two large AD cohorts, we identified 57 CSF proteins associated with depressive symptoms, including several proteins that have not previously been linked to depression in the context of AD. Pathway enrichment analysis revealed that cell adhesion/inflammation, synaptic dysregulation/signaling, and neurogenesis were among the enriched biological pathways related to depressive symptoms. In subjects with cerebral amyloid pathology, additional enriched pathways associated with depressive symptoms were mainly part of cholesterol transport and metabolism. None of the identified proteins were associated with GDS scores at FU visits, nor were their changes from baseline. The identified alterations associated with depressive symptoms indicate underlying pathophysiological processes that are also linked to AD pathology.[Table alz71054-tbl-0002]

Among the 946 CSF common proteins considered, 57 proteins were associated with depressive symptoms in both cohorts, with higher levels of 48 proteins and lower levels of 8 proteins consistently associated with higher GDS scores. Only a few studies previously investigated CSF proteome changes in relation to depressive symptoms. These studies investigated small samples of adults with MDD in midlife.^35^ One such study revealed 161 dysregulated proteins in 40 MDD patients,^15^ of which 12 were associated with depressive symptoms in our study in both cohorts: APP, ATPase H+ transporting accessory protein 1 (ATP6AP1), biotinase (BTD), calsyntenin‐1 (CLSTN1), CST3, MAG, neurofascin (NFASC), opioid‐binding protein/cell adhesion molecule (OPCML), RELN, testican‐1 (SPOCK1), transmembrane protein 59 like (TMEM59L), and WAP four‐disulfide core domain 2 (WFDC2). Notably, APP and CST3 were also reported to be upregulated in another study involving 12 MDD patients.^36^ To our knowledge, no previous study has investigated CSF proteome alterations related to depressive symptoms in older people or in the context of AD. Using untargeted proteomics in two AD cohorts, our results indicate that some alterations observed in MDD patients in midlife are also related to depressive symptoms in older people. Furthermore, our study identified several new CSF proteins associated with depressive symptoms that may be specific for cognitive decline and AD. Considering that these alterations were observed in both cohorts, these findings warrant further investigations.

We identified 1 of the 57 proteins in EMIF‐AD MBD and 5 of the 57 proteins in ADC as also being associated with the presence of depression, defined as a GDS score > 4; however, these associations did not remain significant after FDR correction. The results should be interpreted with caution, as only ≈ 20% of participants were classified as having depression, resulting in limited statistical power. Furthermore, a part of the participants in the “no depression” group still had mild depression symptoms as indicated by a GDS score between 1 and 4. It is important to note that these proteomic datasets were designed primarily to investigate AD pathophysiology, and individuals with more severe depressive symptoms were excluded. Therefore, the sample was not optimally designed to address categorical depression. Overall, these results suggest that the identified proteomic alterations are more closely related to overall depressive symptom severity rather than strictly to the presence of depression.

We identified cell adhesion, synaptic dysfunction, and neurogenesis as the most consistently enriched biological pathways. These pathways have also been found to be involved in the pathogenesis of major depression^37^ and in AD.^38^, ^39^, ^40^ Cell adhesion molecules (CAMs) play important roles in inflammation and the immune response, and their dysregulation may lead to pathological inflammatory processes.^41^ Several studies have revealed an association between systemic and central nervous system inflammation and depression.^42^, ^43^, ^44^, ^45^ Accordingly, targeting inflammation could represent a therapeutic strategy for patients presenting depressive symptoms, including patients with AD, with an underlying inflammatory component.^46^ Synaptic dysfunction has been proposed to play an important role in depression through the modulation of long‐term potentiation and long‐term depression.^47^ Decreased neurogenesis has been linked to psychiatric symptoms, including depression.^45^ Stressful experiences have been shown to negatively impact neurogenesis especially in the hippocampus.^48^ However, the use of antidepressants may stimulate neurogenesis.^49^ Our study applies an untargeted, data‐driven CSF proteomic approach to identify pathophysiological processes involved in depressive symptoms in older individuals with cognitive decline and AD, thereby extending previous proteomic research performed in younger populations and in plasma‐based studies. Given that these pathways are known to be also implicated in the AD pathophysiology, potential treatment strategies targeting these pathways to reduce depressive symptoms may be particularly beneficial in patients with AD.

Several studies have investigated possible associations between markers of AD pathology and depressive symptoms, including early clinical stages of AD, and reported no or weak associations.^7^, ^8^, ^34^, ^50^, ^51^ To our knowledge, no previous study has investigated CSF proteomic profiles of depressive symptoms while simultaneously considering markers of AD pathology. While interaction analyses revealed no moderating effect of amyloid status on any of the identified proteins, stratified analyses revealed additional proteins associated with depressive symptoms in amyloid‐positive individuals, including several lipoproteins (APOA1, APOC1, APOC3, and APOF) involved in cholesterol transport and lipid metabolism.

APP levels were found to be dysregulated in MDD patients in two previous studies.^15^, ^36^ Importantly, neither of these studies provided information regarding the presence of AD pathology, limiting the interpretability of the findings. In our study, APP showed a strong association with depressive symptoms in amyloid‐positive individuals and high interconnectivity with other proteins related to depressive symptoms (Figure 3C). APP plays a central role in the pathogenesis of AD by generating Aβ peptides.^52^ APP is also linked to inflammation,^53^ synaptic dysfunction,^54^ and neurogenesis,^55^ all of which have also been proposed to contribute to the pathogenesis of depression.^46^, ^47^, ^48^ Inflammatory cytokines may in turn influence APP processing, increasing Aβ production and neuroinflammation, which may further contribute to the manifestation of depressive symptoms.^56^, ^57^, ^58^ Additionally, cholesterol dysregulation can disrupt APP processing and contribute to AD pathology and cognitive decline.^59^, ^60^, ^61^ It has also been linked to neuropsychiatric symptoms, including depression and anxiety.^62^, ^63^ Together with previous evidence,^64^, ^65^ our results suggest that APP and lipid metabolism not only are part of AD pathology but also may contribute to the pathogenesis of depressive symptoms. Figure S4 in supporting information summarizes the proposed shared mechanisms between AD pathology and depression. More research is necessary to better understand these relationships and to identify possible intervention targets.

In addition, previous transcriptomic approaches using single‐nucleus RNA sequencing (snRNA‐seq) of brain tissue in adults with MDD^66^, ^67^ and in AD^68^ reported dysregulated biological processes that align with our CSF findings. In MDD, snRNA‐seq studies reported oligodendrocyte lineage cells and excitatory neurons, with differentially expressed genes affecting cell signaling and synaptic plasticity pathways.^66^, ^67^ Furthermore, a cross‐disorder transcriptomic analysis across neurodegenerative and psychiatric conditions (*n* = 4711 *post mortem* brain samples) reported shared pathways, including microglial activation, inflammation, synapse development, and synaptic plasticity, supporting potential shared mechanisms across neuro‐psychiatric disorders.^69^ However, it must be considered that the relationship between transcriptomics and protein levels is complex due to post‐transcriptional regulation and protein turnover and may not always correlate.

None of the identified proteins were associated with GDS scores at FU visits or with their changes from baseline. This finding does not support the hypothesis that pathophysiological alterations in depressive symptoms are also associated with persisting depressive symptoms over time.^13^ However, in both cohorts, GDS scores of participants improved over time, especially in participants with more pronounced depression symptoms (GDS > 4) at baseline. These participants may have received clinical interventions such as antidepressant treatment, psychotherapy, or psychosocial support. However, data on such interventions were not available in this study.

We acknowledge several limitations of our study. Individuals with severe neuropsychiatric symptoms were not included, as the cohorts were built to investigate cognitive decline first, and severe psychiatric symptoms may have interfered with the results of the cognitive assessments. Another limitation is the unavailability of data on antidepressant or other psychoactive medication use, which may influence protein expression and symptom severity and could confound the observed associations. While this is the largest study using CSF proteomics in relation to depressive symptoms thus far, the study was performed in AD cohorts, limiting generalizability. Further studies in cohorts without a focus on AD or including a large number of amyloid‐negative participants could help us to better understand biological alterations related to the manifestation of depressive symptoms in older people with and without amyloid pathology. Because of our stringent approach to selecting proteins only if they were consistently associated with depressive symptoms in both cohorts, we may have missed potentially relevant associations of proteins with depressive symptoms when they had weaker effect sizes. However, this approach ensured that the findings presented are robust and replicated across two independent cohorts. Additionally, considering available CSF amyloid markers, we were able to investigate proteome alterations associated with depressive symptoms in the context of amyloid pathology. Another strength of the present study was the use of CSF proteomics, whereas previous studies have focused mostly on blood protein alterations. CSF is in closer proximity to the brain, and CSF protein levels may better reflect pathophysiological changes in the central nervous system related to depression. Future studies measuring both CSF and blood proteomic changes in relation to depressive symptoms could help to better understand the relationship between blood and CSF alterations and facilitate the development of easily accessible blood‐based biomarkers.

## 5 CONCLUSION

This study used untargeted CSF proteomics to investigate depressive symptoms in older adults in the context of AD. We identified 57 proteins linked to depressive symptoms across two large independent memory clinic cohorts. Pathway enrichment analysis revealed key biological processes, including cell adhesion/inflammation, synaptic dysregulation/signaling, and neurogenesis, underscoring their relevance in depression. Proteome alterations related to amyloid pathology and cholesterol metabolism may reflect shared biological mechanisms of AD and depression. These findings warrant further investigation in more diverse cohorts, including more severe depressive syndromes and non–AD‐related depressive symptoms in older people.

## AUTHOR CONTRIBUTIONS

Miriam Rabl, Willem L. Hartog, Pieter Jelle Visser, Betty M. Tijms, and Julius Popp designed the study and carried out the statistical analysis. Miriam Rabl, Betty M. Tijms, and Julius Popp drafted the manuscript. Betty M. Tijms and Julius Popp supervised the work. All the authors provided critical review, edited the manuscript, and approved the final manuscript.

## CONFLICT OF INTEREST STATEMENT

H.Z. has served on scientific advisory boards and/or as a consultant for Abbvie, Acumen, Alector, Alzinova, ALZpath, Amylyx, Annexon, Apellis, Artery Therapeutics, AZTherapies, Cognito Therapeutics, CogRx, Denali, Eisai, Enigma, LabCorp, Merck Sharp & Dohme, Merry Life, Nervgen, Novo Nordisk, Optoceutics, Passage Bio, Pinteon Therapeutics, Prothena, Quanterix, Red Abbey Labs, reMYND, Roche, Samumed, ScandiBio Therapeutics AB, Siemens Healthineers, Triplet Therapeutics, and Wave; has given lectures sponsored by Alzecure, BioArctic, Biogen, Cellectricon, Fujirebio, LabCorp, Lilly, Novo Nordisk, Oy Medix Biochemica AB, Roche, and WebMD; is a cofounder of Brain Biomarker Solutions in Gothenburg AB (BBS), which is a part of the GU Ventures Incubator Program; and is a shareholder of MicThera (outside submitted work). R.V. has served on advisory boards and/or as a consultant for AC Immune, Novartis, Roche, and Prevail. J.P. has served on scientific advisory boards and/or as a consultant for Eisai, OM Pharma, Lilly, Schwabe Pharma, and Roche. W.v.d.F. is a consultant for the Oxford Health Policy Forum CIC, Roche, Biogen MA Inc., Eisai, Eli‐Lilly, Owkin France, and Nationale Nederlanden Ventures. B.M.T. provided scientific advice to Novo Nordisk. All other authors have nothing to disclose. Author disclosures are available in the Supporting Information.

## CONSENT STATEMENT

The institutional review boards of all the participating institutions approved the procedures for this study. Written informed consent was obtained from all participants or surrogates prior to inclusion.

## Supporting information



Supporting Information

Supporting Information

Supporting Information

## Data Availability

The proteomics data of the EMIF‐AD MDB cohort have been deposited to the ProteomeXchange Consortium via the PRIDE partner repository^70^ with the dataset identifiers PXD019910 and https://doi.org/10.6019/PXD019910. All other data are available from the corresponding author upon reasonable request.
