# Supplementary material for: Cerebrospinal fluid proteome alterations related to depressive symptoms in cognitive decline and Alzheimer's disease
Source: Alzheimers Dement. 2025 Dec 26;21(12):e71054. doi: 10.1002/alz.71054 (PMC12741918; doi:10.1002/alz.71054)
Supplement: Supplementary file 2 — Supporting Information [file ALZ-21-e71054-s001.docx]

**Supplementary Figures S1-S4**

**Cerebrospinal fluid proteome alterations related to depressive symptoms in cognitive decline and Alzheimer’s disease**

**Figure S1. Biological processes and protein‒protein interaction network associated with depressive symptom**

***a*** *Bar plot showing the frequency of different biological processes related to the selected proteins associated with depressive symptoms according to the Panther database. A total of 422 proteins in the ADC, 177 proteins in the EMIF cohort, and 57 proteins overlapping across cohorts were compared.*

***b*** *Protein‒protein interaction network of the 57 selected proteins related to depression identifies via the STRING network analysis tool. Five proteins were added as predicted functional partners: APCS, CNTNAP1, CNTN2, NGFR,and NRCAM.*

**Figure S2. Proteins and pathways associated with depressive symptoms stratified according to sex**

******

*Selection of proteins associated with depressive symptoms in femalse and males. Enriched KEGG and GO pathways for males are shown.*

**Figure S3. Proteins and pathways associated with depressive symptoms stratified according to APOE ε4 status**

*Selection of proteins associated with depressive symptoms in APOE ε4 noncarriers and carriers. Enriched KEGG pathways in APOE ε4 carriers are shown.*

**Figure S4. Hypothetical shared mechanisms between amyloid precursor protein and depression symptoms**

***
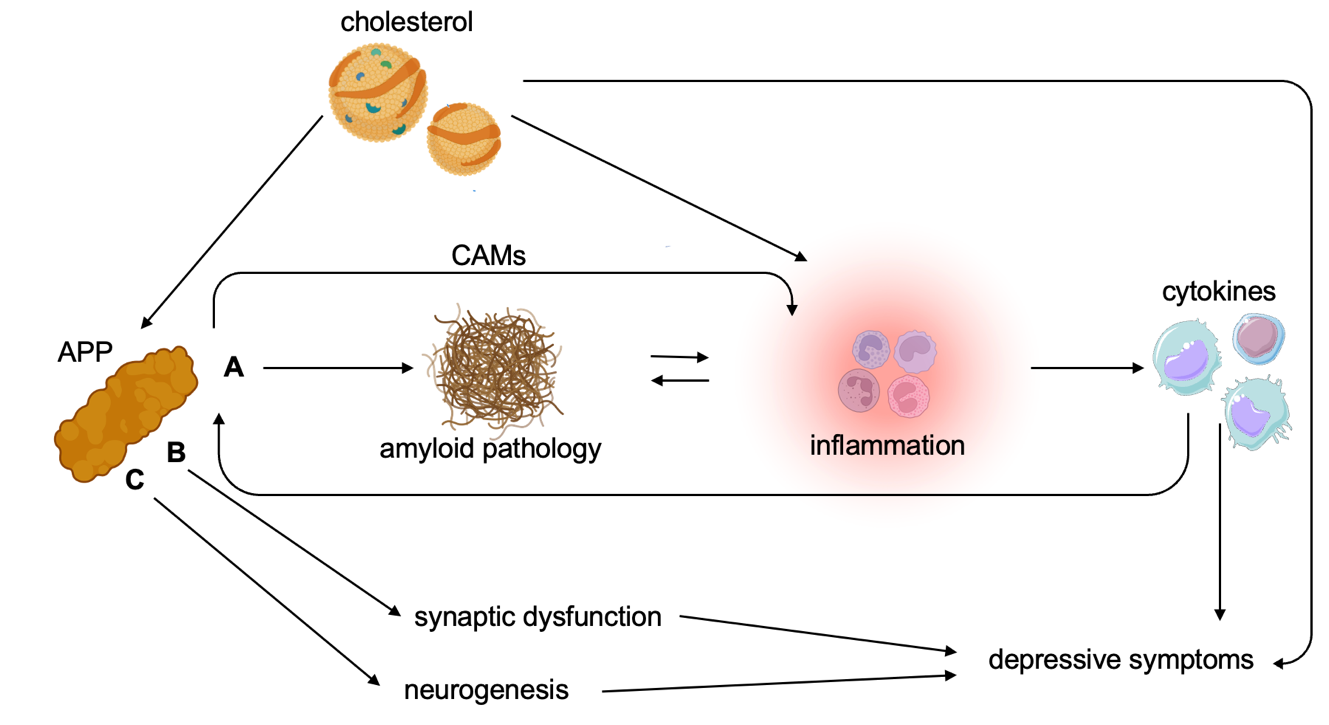
***

***a*** *APP processing leads to amyloid pathology and inflammation processes, which involve CAMs. Inflammation releases cytokines which themselves influence APP processing and increase Aβ production. This feedback loop may further perpetuate inflammation and depressive symptoms.* *APP leads to both synaptic dysfunction* ***(b)*** *and impaired neurogenesis* ***(c)****, which are known to be involved in the pathophysiology of depression. Alterations in cholesterol metabolism influence both APP production and inflammation, potentially also affecting depression. AD, Alzheimer’s disease; APP, amyloid precursor protein; CAMs, cell-adhesion molecules.*
